# Instrument-based Tests for Measuring Anterior Chamber Cells in Uveitis: A Systematic Review

**DOI:** 10.1080/09273948.2019.1640883

**Published:** 2019-08-16

**Authors:** Xiaoxuan Liu, Ameenat L. Solebo, Livia Faes, Sophie Beese, Tasanee Braithwaite, Matthew E. Round, Jesse Panthagani, Aditya U. Kale, Thomas W. McNally, Didar Abdulla, Pearse A. Keane, David J. Moore, Alastair K. Denniston

**Affiliations:** 1 Academic Unit of Ophthalmology, Institute of Inflammation & Ageing, College of Medical and Dental Sciences, University of Birmingham, Birmingham, UK; 2 NIHR Biomedical Research Centre for Ophthalmology, Moorfields Eye Hospital NHS Foundation Trust and UCL Institute of Ophthalmology, London, UK; 3 Institute of Child Health, University College London, London, UK; 4 Department of Ophthalmology, Cantonal Hospital Lucerne, Lucerne, Switzerland; 5 Institute of Applied Health Research, College of Medical and Dental Sciences, University of Birmingham, Birmingham, UK; 6 Ophthalmology Department, University Hospitals Birmingham NHS Foundation Trust, Birmingham, UK; 7 Moorfields Eye Hospitals NHS Foundation Trust, London, UK; 8 Sandwell and West Birmingham Hospitals NHS Trust, Birmingham, UK; 9 Centre for Rare Diseases, Institute of Translational Medicine, Birmingham Health Partners, Birmingham, UK

**Keywords:** Anterior chamber cells, aqueous humor, aqueous humour, diagnostic test, laser flare-cell photometry, optical coherence tomography, systematic review, uveitis

## Abstract

**Purpose:**

New instrument-based techniques for anterior chamber (AC) cell counting can offer automation and objectivity above clinician assessment. This review aims to identify such instruments and its correlation with clinician estimates.

**Methods:**

Using standard systematic review methodology, we identified and tabulated the outcomes of studies reporting reliability and correlation between instrument-based measurements and clinician AC cell grading.

**Results:**

From 3470 studies, 6 reported correlation between an instrument-based AC cell count to clinician grading. The two instruments were optical coherence tomography (OCT) and laser flare-cell photometry (LFCP). Correlation between clinician grading and LFCP was 0.66–0.87 and 0.06–0.97 between clinician grading and OCT. OCT volume scans demonstrated correlation between 0.75 and 0.78. Line scans in the middle AC demonstrated higher correlation (0.73–0.97) than in the inferior AC (0.06–0.56).

**Conclusion:**

AC cell count by OCT and LFP can achieve high levels of correlation with clinician grading, whilst offering additional advantages of speed, automation, and objectivity.

Uveitis, an umbrella term describing inflammatory ocular conditions, is a significant cause of blindness worldwide.^1–3^ Anterior uveitis describes inflammation affecting the anterior chamber (AC), which is predominantly characterized by AC cells and flare, where disruption of the blood-aqueous barrier results in leakage of inflammatory blood constituents into the aqueous humor.

Detection and monitoring of disease activity is crucial for rationalizing medical therapy, which is particularly important because therapeutic interventions for uveitis carry risks of significant adverse ocular and systemic side effects; these include cataract raised intraocular pressure and opportunistic infection. The Standardization of Uveitis Nomenclature (SUN) Working Group proposed the now preferred clinical AC cell grading system.^4^ In this, an observer aims a 1 mm^2^ light beam through the AC and counts the number of illuminated cells visible. The cell count is then placed into one of six grades in the SUN grading system (Table 1). Prior to SUN, a number of alternative systems existed that quantified cells in a similar way.^5–9^

**Table 1.** Clinician grading scales used in each study.

		Previously published grading systems			Number of cells in each grade, by study					
**Grade**		**SUN**	**Hogan**	**BenEzra**	**Igbre**	**Invernizzi**	**Sharma**	**Li**	**Ohara**	**Tugal-Tutkun**
	**0**	<1	0	<5	<1	<1	<1	0	0	<5
	**0.5**	1–5	-	-	1–5	1–5	1–5	1–5	1–4	-
	**1**	6–15	5–10	5–10	6–15	6–15	6–15	6–10	5–10	5–10
	**2**	16–25	10–20	11–20	16–25	16–25	16–25	11–20	11–30	11–20
	**3**	26–50	21–50	21–50	26–50	26–50	26–50	21–50	31+	21–50
	**4**	50+	50+	50+	50+	50+	50+	50+	-	50+
	**5**	-	-	hypopyon	-	-	-	-	-	hypopyon

**Table 2.** Study Characteristics.

Author	Year	Study Design	No. of participants	No. of eyes	Gender, no. of eyes (%)	Mean age, years (range)	Aetiological classification, no. of eyes (%)
Ohara	1989	Prospective	124	127	44 (35%) male 80 (65%) female	NR (12–76)	Sarcoidosis 53 (43%), Behcet’s 14 (11%), VKH 6 (5%) Bilateral ARN 3 (2%), other 14 (11%), unknown 34 (27%)
Tugal-Tutkun	2008	Prospective	232	153	124 (53%)male 108 (47%) female	Active ocular Behcets, 28 (NR) Inactive ocular Behcets, 29 (NR) Non-ocular Behcet’s group, 31 (NR) Healthy control, 27 (NR)	Active ocular Behcets 54 (35%) Inactive ocular Behcets 53 (23%) Non-ocular Behcets 78 (34%) Health control 47 (20%)
Li	2013	Prospective	35	66	NR	NR	Non-granulomatous 30 (39%) Granulomatous 12 (16%) Quiescent 16 (21%) Healthy control 19 (25%)
Igbre	2014	Retrospective	41	78	12 (29%) male 29 (71%) female	48 (10–83)	Non-granulomatous anterior uveitis 9 (23%), sarcoidosis 6 (16%), HLA-B27 5 (12%), Panuveitis 4 (10%), intermediate uveitis 3 (7%), granulomatous anterior uveitis 2(6%), uveitis glaucoma hyphema syndrome 1 (2%), BCR 1 (2%), HSV 1 (2%), JIA 1 (2%), multifocal choroiditis 1 (2%), scleritis 1 (2%), herpetic keratouveitis 1 (2%), pars planitis 1 (2%), VKH 1 (2%), sympathetic ophthalmia 1 (2%), unknown 2 (6%),
Sharma	2015	Prospective	76	114	Single line scan: 16 (32%) male 34 (68%) female Volume scan: 8 (27%) male 22 (73%) female	43 (12–94)	Line scan: Idiopathic 34 (41%), HLA B27 17 (21%), JIA 12 (15%), Sarcoidosis 10 (12%), VKH 6 (7%), HSV 2 (2%), Behçet’s 2 (2%) Volume scan: Idiopathic 10 (32%), HLA-B27 7 (23%), JIA 7 (23%), Sarcoidosis 4 (13%), VKH 1 (3%), Psoriatic arthritis 2 (6%).
Invernizzi	2017	Prospective	122	237	NR	Healthy controls 42 (NR) Inactive uveitis 43 (NR) Active uveitis 45 (NR)	Healthy controls 70 (30%) Inactive uveitis 97 (40%) Active uveitis 70 (30%)
NR – not reported, ARN - acute retinal necrosis, VKH - Vogt-Koyanagi-Harada, BCR - birdshot chorioretinopathy, HSV - herpes simplex virus, JIA - juvenile idiopathic arthritis).							

Author	Index test	Manufacturer and model	Image acquisition settings	Position scanned	Area	Volume	Image analysis software	Level of automation
Ohara	LFCP	FC-1000, KOWA (Kowa Company, Ltd, Tokyo, Japan)	Final reading is the average of 5 repeated readings.	NR	NA	0.075 mm^3^	None	Semi-automated
Tugal-Tutkun	LFCP	FC-2000, KOWA (Kowa Company, Ltd, Tokyo, Japan)	Seven readings are obtained. The highest and lowest values are discarded and mean of the remaining five readings is the final reading.	NR	NA	0.5mm^3^	None	Semi-automated
Li	OCT	Time domain OCT prototype system (Carl Zeiss Meditec Inc., Dublin, CA). A time-domain OCT system with an axial resolution of 17 µm and transverse resolution of 45 µm.	Two concentric circular scans with diameters of 2 and 4 mm. Inner and outer circular scans consisted of 256 and 512 axial scans, respectively. Each AC grading scan was divided into three regions: central (inner circular scan), superior (superior semicircle of the outer circular scan), and inferior (inferior semicircle of the outer circular scan).	Inferior, central and superior	2 concentric circular scans of 2 and 4 mm diameter	NR	Unspecified custom software developed by authors	Automated
Igbre	OCT	AS-OCT (Visante OCT, Zeiss Meditec Dublin, CA	Between 4 and 8 high-resolution corneal cross-sectional scans (10mm across each).	NR	4 to 8 lines of 10 mm width	NA	Image pre-processing 30% desaturation (Photoshop; Adobe Systems, San Jose, CA)	Manual
Sharma	OCT	The RTVue-100/CAM (Optovue Inc., Fremond, CA) with corneal adapter	Line scan: 6mm single B-scan at the central cornea. 3D volume scan: 6mm^3^ consisting of 512 B-scans each with 128 A-scans.	Central	One 6 mm line	Line scan: NA 3D volume scan: 6mm^3^	Custom software developed by authors: *ImageIQ* (Cleveland Ohio)	Automated for 3D scans, manual for line scan
Invernizzi	OCT	Casia SS-1000 OCT device (Tomey Corporation, Nagoya, Japan).	Two 6mm cross-sectional scans with a depth of 2048 A/B scans	Central	Two 6mm lines	NA	Built in software (Tomey Link Exam Viewer Version 7F.2)	Manual
NR – not reported, NA – not applicable, LFCP – laser flare-cell photometry, OCT – optical coherence tomography, SS – Swept source								

Author	Index test	Clinical Grading System	Observer	No. eyes in each clinical grade				
Ohara	LFCP	Unspecified	NR for index test	NR				
			One observer for reference test					
Tugal-Tutkun	LFCP	BenEzra	One observer for index test	Grade 0 (7)				
			One observer for reference test	Grade 0.5 to 2 (26)*				
			(different observers)	Grade 3–4 (17)*				
				Grade 5 (hypopyon) 0				
Li	OCT	Modified Hogan	NR for index test	Grade 0.5 (6 non-granulomatous, 4 granulomatous)				
			One observer for reference test	Grade 1 (10 non-granulomatous, 3 granulomatous)				
				Grade 2 (2 non-granulomatous, 2 granulomatous)				
				Grade 3 (8 non-granulomatous, 2 granulomatous)				
				Grade 4 (4 non-granulomatous, 0 granulomatous)				
Igbre	OCT	SUN	One observer for index test	Grade 0 (22)				
			One observer for reference test	Grade 0.5 (20)				
			(same observer)	Grade 1 (13)				
				Grade 2 (13)				
				Grade 3 (1)				
				Grade 4 (0)				
Sharma	OCT	SUN	One observer for index test	Grade 0 (24)				
			One of two senior uveitis specialists for reference test	Grade 0.5 (5)				
				Grade 1 (16)				
				Grade 2 (15)				
				Grade 3 (11)				
				Grade 4 (12)				
Invernizzi	OCT	SUN	One observer for index test	Grade 0 (6)				
			NR for reference test	Grade 0.5 (24)				
				Grade 1 (19)				
				Grade 2 (15)				
				Grade 3 (3)				
				Grade 4 (3)				
*Tugal-Tutkun et al. reported number of eyes in grades 0.5–2 and 3–4 combined.								

Author	Index test	Clinical Grading System	No. eyes included in correlation analysis n (description)	Correlation Coefficient (95% CI) Spearman’s *r*				
Ohara	LFCP	Not specified	NR	*r* = 0.66 (NR)				
Tugal-Tutkun	LFCP	BenEzra	54 (active uveitis group)	*r*= 0.87 (NR)				
Li	OCT	Modified Hogan	30 (Non-granulomatous group)	Non-granulomatous:				
			12 (granulomatous group)	Central AC *r*= 0.73 (0.55–0.88)				
				Superior AC *r*= 0.75 (0.56–0.88)				
				Inferior AC *r*= 0.56 (0.36–0.81)				
				Granulomatous:				
				Central AC *r* = 0.74 (CI NR; estimated as 0.29–0.92*)				
				Superior AC *r*= 0.73 (CI NR; estimated as 0.27–0.92*)				
				Inferior AC *r*= 0.06 (CI NR; estimated as −0.53–0.61*)				
Igbre	OCT	SUN	69 (all included subjects)	*r*= 0.74 (0.62 to 0.83)				
Sharma	OCT	SUN	83 eyes (single line scan group)	Single line scan *r* = 0.97 (CI NR; estimated as 0.95–0.98*)				
			31 eyes (volume scan group)	Manual volume scan *r*= 0.78 (0.60–0.90)				
				Automated volume scan *r*= 0.75 (0.55–0.88)				
Invernizzi	OCT	SUN	70 (active uveitis group)	*r* = 0.94 (0.91–0.96)				

Multiple limitations of this system are recognized. First, it is prone to bias due to reliance on subjective estimation of an observer. Although instructions dictate that cell counting should be carried out in one moment in time, in reality, this is a near-impossible task, especially at higher grades where cell counts exceed 30–40 cells/mm^2^. Second, the SUN grading system uses a non-linear, non-continuous scale with large steps between grades. Changes in inflammatory activity within one grade may go undetected, especially in the higher grades. Third, it relies upon the presence of an ophthalmic clinician trained in slit-lamp biomicroscopy, and therefore limits disease monitoring to a hospital setting. Consequently, delivery of uveitis care in other health-care settings such as remote screening and community-based monitoring has not been feasible.

Instrument-based techniques such as laser flare-cell photometry (LFCP), and more recently anterior segment optical coherence tomography (AS-OCT), have shown potential for objectively quantifying AC cells. LFCP became available in 1988 and uses the light scattering properties of AC particles to quantify the concentration of inflammatory materials in the aqueous humor. It has been primarily validated as a tool for measuring AC flare,^10^ the cloudy appearance given to the aqueous during inflammation, however several models also have the ability to count AC cells. AS-OCT provides cross-sectional scans of the AC and can capture cells in aqueous humor as hyper-reflective dots. Given the drive towards objective, quantitative assessment of disease status, a systematic examination of the evidence for such technologies is timely.^11,12^ This review aims to identify all instrument-based tools for counting AC cells and evaluate their correlation with clinician grading systems.

## METHODS

This review was reported according to the Preferred Reporting Items for Systematic Reviews and Meta-Analysis (PRISMA) statement.^13^ The methodology was specified in advance and protocol registered with PROSPERO (CRD42017084156).^14,15^

### Eligibility Criteria

We included studies that described one or more instrument-based methods for counting AC cells in patients with uveitis (index tests) in comparison to a clinician grading system (through slit-lamp examination). We also included studies reporting test reliability (e.g., intra or inter-observer reliability and/or repeatability). We did not place restrictions on age, gender, ethnicity, underlying etiology or disease activity status. Animal studies and studies involving only healthy participants, single case reports, commentaries, opinion articles, and pictorial articles were excluded.

The primary outcome was the level of correlation between index tests and clinician grading. The secondary outcome was intra/inter-observer reliability and repeatability of the index test.

### Search Methods for Identifying Studies

We combined free text terms and index terms reflecting the pathological finding of interest ‘cells’ and ‘anterior chamber’ or ‘aqueous humor’, and the disease context ‘uveitis’. The search strategy was adapted to match the index terms in different databases (**supplementary materials**). Database searches were carried out in MEDLINE, Embase, Cochrane Controlled Register of Trials (CENTRAL), Centre for Reviews and Dissemination Database (Health Technology Assessments and the Database of Abstracts and Reviews of Effects), Clinicaltrials.gov, WHO International Clinical Trials Registry Platform (ICTRP portal), British Library’s ZETOC, Conference Proceedings Citation Index (Web of Science), British Library Ethos, ProQuest and OpenGrey. We searched all databases from inception to 22 March 2018, with no date or language restrictions. We manually searched citations of review articles and included studies to identify additional relevant articles.

### Study Selection

Two reviewers independently screened studies at each stage. Disagreements were resolved through discussion and input from a third reviewer.

### Data Collection

Two reviewers extracted data independently using a pre-specified data extraction sheet. The data included population characteristics (number of participants, gender, age, underlying etiology), index test characteristics (technology, manufacturer, model, image acquisition settings, area sampled and software automation), clinician grading (name of grading system used, number of patients in each grade) and outcome (correlation coefficient, inter/intra-observer reliability). Cell counting analysis was recorded as fully automated, semi-automated or manual. For the clinician grading, we extracted how each grade was defined and whether any modifications were made to validated clinical grading systems. We contacted three authors for further information^16–18^, all of whom responded and one provided further data (confidence intervals) which was not reported in the original paper.^16^

### Risk of Bias Assessment

Two reviewers independently assessed risk of bias using the Quality Assessment of Diagnostic Accuracy Studies tool (QUADAS-2).^19^ We adapted each element in QUADAS-2 to address the review question. Specifically, we explored potential sources of bias arising from the index test and clinician grading procedures: whether the test protocols were determined *a priori* and standardized for all participants, and whether observers were blinded to test measurements.

### Data Synthesis and Analysis

For each outcome, studies were grouped by index test technology and then by choice of clinician grading tool. For each technology, we tabulated the evidence and provided a narrative synthesis. Where authors modified clinician grading systems, these were considered separately from the validated versions (Table 2). Where confidence intervals for correlation coefficients were not reported, we estimated them using sample size and correlation coefficient and presented this on a forest plot. All statistical analysis was performed using Stata Statistical Software (Release 15. College Station, TX: StataCorp LP.)

## RESULTS

### Results of the Search

**Figure 1.**
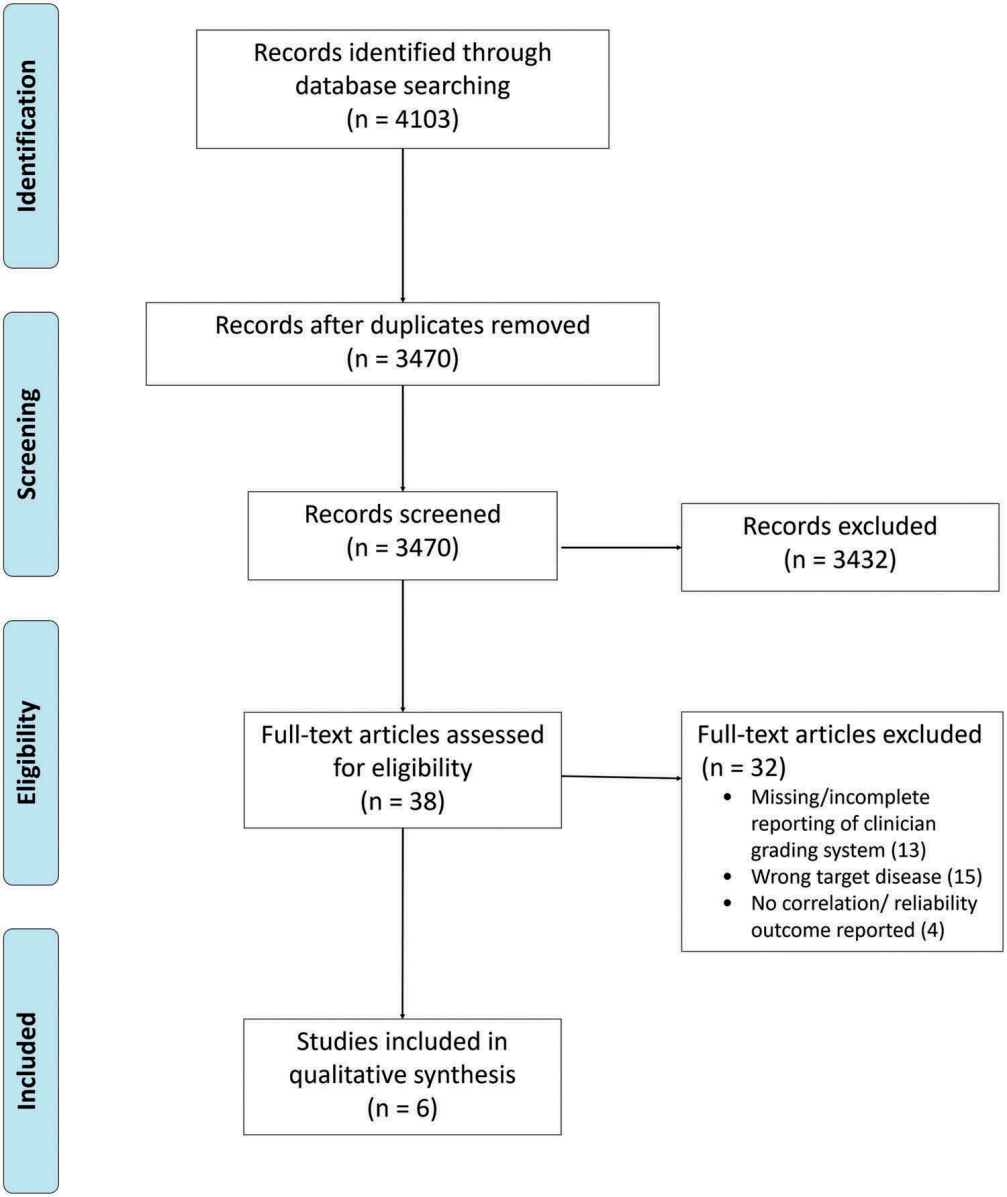
PRISMA flow diagram.

The study selection process is summarized in the PRISMA flow diagram (Figure 1). The searches from database conception to 22 March 2018 yielded 3470 bibliographic records after de-duplication. Of these, 3432 were excluded upon screening of titles and abstracts. The large number of exclusions is due to the unrestrictive nature of our search strategy, which did not specify any index test terms, and the small number of published studies that made comparisons between an index test and clinician grading. The remaining 38 articles were obtained in full text for further scrutiny and a further 32 articles were excluded. The reasons for exclusion were missing or incomplete reporting of clinician grading system (n = 13), the target disease not being uveitis (n = 15) and no correlation/reliability outcome reported (n = 4). Six unique studies met the eligibility criteria and were included (Table 1).

### Methodological Quality of the Included Studies

Using QUADAS-2, one study was identified as having unclear risk of bias for patient selection due to the exclusion of patients with posterior synechiae **(supplementary figure)**, which is known to affect LFCP readings.^20^ Another study had an unclear risk of bias in the index test domain as it was unclear whether observers were blinded to the clinician grading.^21^ One study had a high risk of poor applicability due to patient selection, as only patients with Behcet’s disease were included.^20^ We graded all studies as having unclear risk of bias in the reference test domain due to previously mentioned concerns around the reliability of subjective clinician grading.

### Patients’ Characteristics and Design Features

The six studies enrolled 775 eyes (from 630 subjects) and dated from 1989 to 2017.^16–18,20–22^ Participants were recruited prospectively in all studies, except for Igbre et al. who used existing clinical data. All comparisons between index test and clinician grading were done in a cross-sectional manner. Gender was reported in 5 out of 6 studies, in which 47.8% (n = 301) participants were male. The mean age was 45.2 years (range 27.0–81.0 years). Four studies included mixed etiologies, one study did not report underlying etiology,^16^ and one included only Behcet’s disease patients.^20^ The underlying etiologies across all studies included non-granulomatous uveitis, sarcoidosis, HLA-B27-associated uveitis, unspecified panuveitis, unspecified intermediate uveitis, pars planitis, acute retinal necrosis, granulomatous uveitis, juvenile idiopathic arthritis, Behcet’s disease, multifocal choroiditis, sympathetic ophthalmia, Vogt-Koyanagi-Harada disease, uveitis glaucoma hyphema syndrome, birdshot chorioretinopathy, herpes simplex virus-associated uveitis, herpetic keratouveitis, idiopathic uveitis and unknown cause. Three studies included healthy controls,^16,17,20^ but only analyses where uveitis patients separately reported were included in the correlation analyses of this review.

### Clinical Grading Systems

Three studies used the SUN grading system as a comparator,^16,18,22^ one study used the scoring system described by BenEzra et al. in 1991,^9^ one study used a modified version of the 1959 Hogan system,^5,17^ and one study used an unspecified clinical grading system.^21^ Upon contacting the author, the justification for modifying the Hogan grading system was due to the uveitis specialist’s preference.^17^ The differences between the grading systems are outlined in Table 1. Four studies reported the number of subjects with each clinical AC cell grade,^16–18,22^ one study combined grades^20^ (for example, “26 subjects had grades 0.5 to 2”) and one did not report this.^21^ Sources of variation include number of cells seen in each grade (particularly in grades 1 and 2), the addition of a “0.5+” grade in the SUN grading system, the inclusion of a grade 5 to account for presence of hypopyon by the BenEzra system, and the lack of a specified slit beam size in the Hogan and BenEzra systems (SUN grading specifies 1 mm^2^).^5,9^

### Instruments for Measuring AC Cells

Six studies were included for analysis.^16–18,20–22^ All six studies compared the measurements of AC cells on an instrument to a clinical grading system and reported the correlation coefficient. No studies reported reliability for instrument-based grading. We identified two instrument-based technologies for quantifying AC cells: OCT and LFCP.

### Optical Coherence Tomography

Four studies reported correlation between OCT and a clinical grading system.^16-18,22^ Three studies^16,18,22^ used commercially available OCT machines and one used a prototype system.^17^ The scanning protocols (including the scan settings, position, area, and volume scanned) were unique in each study. Li et al. used a time-domain OCT system (Carl Zeiss Meditec Inc., Dublin, CA), with an axial resolution of 17 microns and axial depth of 8 mm, to capture concentric cross-sections of the central AC in 35 uveitis patients (66 eyes). The Visante AS-OCT (Zeiss Meditec Dublin, CA) was used by Igbre et al. to capture 4–8 cross-sectional images in 41 patients (78 eyes), but the chamber position, area and volume scanned were not reported.^22^ Sharma et al. captured line scans and 6 mm^3^ scans at the central cornea using the RTVue-100/CAM (Optovue) in 76 patients (114 eyes).^18^ Invernizzi et al. used the swept source Casia SS-1000 OCT device (Tomey Corporation, Nagoya, Japan) to capture two 6 mm cross-sectional scans of the AC in 167 uveitic eyes and 70 healthy eyes.^16^ Two studies used manual cell counting^16,22^ and one automated this,^17^ whilst the fourth study used both methods.^18^ For the two studies using automated cell counting, algorithms were developed *de novo* for study purposes and are not openly available.^16,18^

### Laser Flare-Cell Photometry

Two studies reported correlation between LFCP and a clinician grading system.^20,21^ In both studies, the LFCPs were manufactured by KOWA (Kowa Company, Tokyo, Japan), but the models differed; FC-1000^20^ and FC-2000.^20^ All flare measurements were calculated automatically using the machine’s built-in function. As per the manufacturer’s recommendations, the observer took several readings, discarded the highest and lowest values, before averaging the final values to derive an average cell count measurement and a standard deviation. Neither study reported the position and area/volume of aqueous scanned.

### Correlation between Index Tests and Clinician Grading Systems

**Figure 2.**
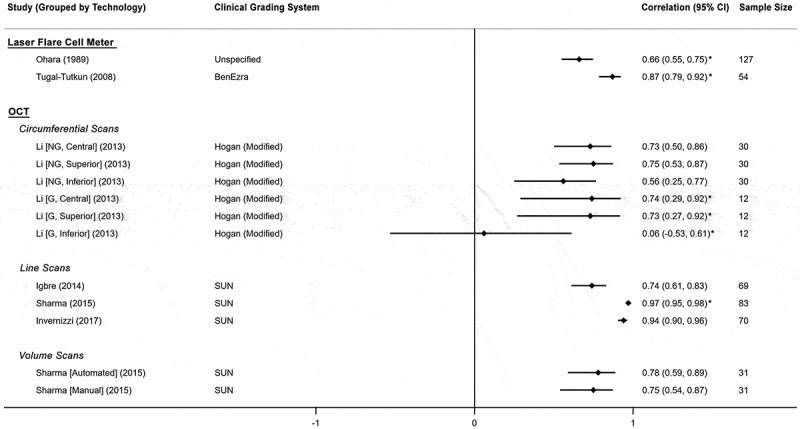
Forest plot of correlation coefficients reported by all included studies between index test measurements versus clinician grading, grouped by index test technology and clinician grading system.

All six studies reported a correlation coefficient between the index test and a clinical grading system, using Spearman’s *r*. The level of correlation between index tests and clinician grading systems is shown using a forest plot (Figure 2).

For the time-domain OCT devices, the correlations were reported to be 0.74 (95% CI 0.62–0.83) in the Visante device (Zeiss Meditec, Dublin, CA),^22^ and up to 0.75 for the prototype Zeiss device, depending on position of the scan^17^ (highest correlation *r* = 0.75 for superior AC and lowest correlation *r* = 0.06 for inferior AC). For the newer spectral-domain or swept source OCT devices, which unlike the time domain models, have a faster acquisition time and maximal axial imaging resolution smaller than the normal range of white cell width (10–17 microns),^23^ higher correlation values were reported (0.97, *p* < .0001 for RTVue-100/CAM, Optovue,^18^ and 0.94, *p* < .0001 for the Casia SS-1000 OCT device, Tomey Corporation.^16^) There was no apparent association between the level of automation of OCT images analysis and the correlation with AC cell count.

OCT can also acquire volume scans by repeating densely placed single line scans. All four studies used single line scans at different positions across the anterior chamber. Sharma et al. additionally compared single line scans to 3D cubic volume scans of 6 mm^3^, and found the single line scans to have higher correlation with the clinical grading than the volume scans (0.94 for single line and 0.74–0.77 for volume scan).^18^

For the LFCP, two studies reported correlation with clinician grading (*r =*0.66^20^ and *r =*0.87^19^). The KOWA FC-2000, which scans a larger volume of aqueous (0.5 mm^3^) than the FC-1000 (0.075 mm^3^), achieved a higher level of correlation (*r =*0.87^19^).

### Study Heterogeneity

There was considerable heterogeneity between the methodology and populations described by the three studies which shared a common comparator (SUN grading).^16,18,22^ Due to the differences in scan acquisition parameters (varying sized scan areas and levels of automation) and distribution of AC cell severity in the study populations (as measured by clinician grading), we did not consider the index test measurements to be directly comparable by meta-analysis.

## DISCUSSION

This is the first systematic review to evaluate instrument-based technologies for counting AC cells in uveitis. We found two technologies for this purpose: OCT and LFCP.

When these technologies were used in a relatively consistent way, with precisely specified measurement and scanning protocols, we found strong correlation with the SUN grading system (*r* = 0.74–0.97). However, the range of correlation for instrument-based measurements versus clinician grading ranged from 0.06 to 0.97. Included studies demonstrated a higher correlation coefficient achieved by OCT than LFCP. However, the inconsistent use of clinical comparators across studies prevented us from making direct comparisons between the technologies.

### Performance and Limitations of Measures of AC Cells

Studies of instrument-based cell counting using OCT versus clinician grading reported correlations of *r* = 0.06–0.97, and for LFCP *r* = 0.66–0.87. The variation in correlations seen between studies of the same platform may arise due to several important factors which may impact instrument-based measures only, human clinical measures only, or both.

#### Factors Affecting Instrument-based Measures

Some variation in the correlation between studies may suggest that not all instrument-based measures of the same technology are equal, and that performance may be affected by the model and technique used. Newer models of OCT have higher resolution (enabling improved discrimination of cells) and faster acquisition time (overcoming the effects of missing or double-counting moving cells).

#### Factors Affecting the Performance of Human-based Clinical Measures

Some variation in the level of correlation may be unrelated to the technology, but rather reflect poor reliability of the clinician-based method. In addition to the well-recognized generic limitations of subjectivity and imprecision,^24,25^ we noted some specific variations in choice of clinician grading systems used across studies. Two studies published after the 2005 SUN Workshop used non-SUN grading systems,^17,20^ and one made a custom modification by adding a 0.5 grade to the pre-SUN Hogan system.^17^ The reasons for this are unclear. It is unlikely that preference for one grading system over another is based on perceptions of superiority, as all clinical grading systems share the same issues around subjectivity. Additional factors that were not always recorded in these studies but are known to impact the reliability of the clinical measure are the experience of the clinician, and number of observers independently scoring each AC.^25^

#### Factors Affecting the Performance Of Both Instrument-based and Human-based Measures

Factors such as patient selection may affect both instrument-based and human measures. For example, including patients with corneal opacity is likely to reduce performance of both measures due to reduced cell discrimination, although there is some evidence it may impact OCT measures less.^26^ Our review found higher levels of correlation for scans involving a smaller area of the central AC. This difference may arise from several factors including:

*Areas sampled*: Li et al. reported weaker correlation between clinical grading and OCT scans taken in the inferior, compared to the middle or superior AC. Li and colleagues suggested there may be an unequal distribution of AC cells from the superior to inferior parts of the AC, and a poorer correlation when comparing the middle AC (captured by clinician grading) and inferior AC (captured by OCT) could be expected. They hypothesized that smaller and lighter cells may be carried by the aqueous circulation to superior parts of the AC, whereas larger and heavier cells in the AC may accumulate at the bottom.^17^

*Acquisition time*: Increased acquisition time may allow floating AC cells to move through the aqueous during successive raster scans resulting in over- or under-counting of cells. Newer OCT models with higher acquisition speeds are unlikely to be affected by this problem; however, time-domain OCT models and various other operator and patient factors (such as poor fixation, opacities, and reflections) may affect time required for scan acquisition.

### Strengths and Limitations of the Review

The strength in this study lies in its systematic approach of reviewing all publications of instrument-based tool for AC cell counting with clinician slit-lamp based grading system. Our search strategy was designed to have high sensitivity for such studies and we searched a broad range of databases, including conference proceedings, dissertation databases, and the grey literature. Our limitations include the assumption that clinician grading, the current gold standard, is an appropriate reference standard for comparison. Our review cannot answer the question of whether an instrument-based measure is more accurate than clinician grading. However, other advantages are apparent, including capture of a larger area of AC and the ability to automate the cell counting process, whilst maintaining a good correlation with the clinician-based method.

### Limitations Due to Gaps in the Evidence

First, due to the small number of included studies and heterogeneity in study design, it was not possible to provide pooled estimates of correlation coefficients. It was also not possible to make direct comparisons between OCT and LFCP due to the non-standardized use of comparators. Second, there would be value in evaluating the techniques across different subgroups to ensure generalisability (i.e., subgroup analysis by different etiological groups, between active and inactive disease and by age group and gender) but none of the current studies reported enough subgroup data. Third, imaging protocols for each study were variable. All studies for OCT acquired line scans, however total area of aqueous captured differed in each study. This might not have been an issue had the number of cells been reported per area/volume of aqueous. However, all studies reported absolute total number of cells observed. Future standardization of the output metrics generated, including cell count per unit of aqueous, is needed. This is essential for reliable comparison between devices such as monitoring a patient over time between different health settings, where multiple devices may be used.

### Clinical Relevance and Impact

This review found that instrument-based tools can achieve high correlation with clinical grading. As discussed, earlier differences in design across studies preclude reliable head-to-head comparison of the two instrument-based techniques, but it is likely that OCT will become the dominant technology for cell counting as the LFCP models offering cell count have been discontinued after the FC-2000. In addition, OCT can be automated and performed without the need for a skilled clinician. Implementing this technology in routine clinical care could potentially offer more quantitative, objective, long-term monitoring of anterior uveitis. These technologies could also permit task-shifting away from a small number of clinical experts to disease monitoring delivered by technicians. This also carries implications for future care delivery models, opening the possibility of remote monitoring and community-based care.

Future studies should consider more explicit reporting of patient, eye and ocular disease characteristics to permit meaningful comparison of methods and devices. Controlled studies, including healthy individuals recruited from the full age range will also be important to capture any non-pathological changes in the permeability of the blood-aqueous barrier, which develops with age. It will also be necessary for devices to demonstrate discriminant validity, correctly identifying AC cellular activity resulting from uveitis, from red blood cells or pigmented iris endothelial cells. Prospective longitudinal studies of patients with quiescent and active inflammation are needed to determine the minimum clinically important difference and inform consensus around diagnostic thresholds.^27^

Based on our review of the literature we would propose that key industry standards that need to be defined in order to support cross-device comparison include: (1) unit of measurement (e.g., cells per mm^3^); (2) volume and location within the AC that is sampled; (3) clear reporting of any custom analysis software, including image pre-processing, thresholds set for identifying image features as cells (such as brightness of pixels), discarding of spurious findings and the degree of manual input required. In addition, all studies that seek to validate such techniques should report: (1) population characteristics (including disease etiology and distribution of disease severity within the cohort); (2) internal validity measures (such as test–retest reliability and inter/intra-rater reliability in the case of non-automated techniques), and (3) confidence intervals for all reported performance metrics.

## Conclusion

Instrument-based technologies such as OCT and LCFP offer objectivity and automation to the assessment of AC cells in uveitis, and in a controlled setting can demonstrate high correlation with the current clinical standard. OCT is likely to become the dominant technology for cell counting and is suitable for the wide-scale deployment that would be necessary for it to become the new standard. However, before this is possible, there is a need for consensus around measurement standards for such instruments that would enable cross-device comparison to support reliable longitudinal measurement for patients in the real world.

## Supplementary Material

Supplemental Material
