# Supplementary material for: Instrument-based Tests for Measuring Anterior Chamber Cells in Uveitis: A Systematic Review
Source: Ocul Immunol Inflamm. 2019 Aug 16;28(6):898–907. doi: 10.1080/09273948.2019.1640883 (PMC7497279; doi:10.1080/09273948.2019.1640883)
Supplement: Supplemental Material [file IOII_A_1640883_SM8263.zip › Figure S1.pdf]

Study	RISK OF BIAS				APPLICABILITY CONCERNS		
	PATIENT SELECTION	INDEX TEST	REFERENCE STANDARD	FLOW AND TIMING	PATIENT SELECTION	INDEX TEST	REFERENCE STANDARD
**Ohara, 1989**	☺	?	?	☺	☺	☺	☺
**Tugal-Tutkun, 2008**	?	☺	?	☺	☹	☺	☺
**Li, 2013**	☺	☺	?	☺	☺	☺	☺
**Igbre, 2014**	☺	☺	?	☺	☺	☺	☺
**Sharma, 2015**	☺	☺	?	☺	☺	☺	☺
**Invernizzi, 2017**	☺	☺	?	☺	☺	☺	☺

☺ Low Risk ☹ High Risk ? Unclear Risk
