# Supplementary material for: Instrument-based Tests for Measuring Anterior Chamber Cells in Uveitis: A Systematic Review
Source: Ocul Immunol Inflamm. 2019 Aug 16;28(6):898–907. doi: 10.1080/09273948.2019.1640883 (PMC7497279; doi:10.1080/09273948.2019.1640883)
Supplement: Supplemental Material [file IOII_A_1640883_SM8263.zip › Supplementary Materials.docx]

# Supplementary materials: MEDLINE Sample Search Strategy

| 1 | Exp Uveitis/ |
| --- | --- |
| 2 | Uveiti*. Ti, ab. |
| 3 | 1 or 2 |
| 4 | Anterior chamber. Ti, ab. |
| 5 | Aqueous humour. Ti, ab. |
| 6 | Aqueous humor. Ti, ab. |
| 7 | 4 or 5 or 6 |
| 8 | Cell*. Ti, ab. |
| 9 | 3 and 7 and 8 |
